# Tumour Microenvironment Stress Promotes the Development of Drug Resistance

**DOI:** 10.3390/antiox10111801

**Published:** 2021-11-11

**Authors:** Nicole A. Seebacher, Maria Krchniakova, Alexandra E. Stacy, Jan Skoda, Patric J. Jansson

**Affiliations:** 1 Department of Oncology, University of Oxford, Oxford OX3 9DU, UK; nicole.seebacher@conted.ox.ac.uk; 2 Department of Experimental Biology, Faculty of Science, Masaryk University, 62500 Brno, Czech Republic; maria.krchniakova@mail.muni.cz; 3 International Clinical Research Center, St. Anne’s University Hospital, 65691 Brno, Czech Republic; 4 Cancer Drug Resistance & Stem Cell Program, School of Medical Science, Faculty of Medicine and Health, The University of Sydney, Camperdown, NSW 2006, Australia; alexandrastacy@gmail.com; 5 Bill Walsh Translational Cancer Research Laboratory, Kolling Institute, Faculty of Medicine and Health, The University of Sydney, St. Leonards, NSW 2065, Australia

**Keywords:** tumour microenvironmental stress, drug resistance, reactive oxygen species, cancer stem cells

## Abstract

Multi-drug resistance (MDR) is a leading cause of cancer-related death, and it continues to be a major barrier to cancer treatment. The tumour microenvironment (TME) has proven to play an essential role in not only cancer progression and metastasis, but also the development of resistance to chemotherapy. Despite the significant advances in the efficacy of anti-cancer therapies, the development of drug resistance remains a major impediment to therapeutic success. This review highlights the interplay between various factors within the TME that collectively initiate or propagate MDR. The key TME-mediated mechanisms of MDR regulation that will be discussed herein include (1) altered metabolic processing and the reactive oxygen species (ROS)-hypoxia inducible factor (HIF) axis; (2) changes in stromal cells; (3) increased cancer cell survival via autophagy and failure of apoptosis; (4) altered drug delivery, uptake, or efflux and (5) the induction of a cancer stem cell (CSC) phenotype. The review also discusses thought-provoking ideas that may assist in overcoming the TME-induced MDR. We conclude that stressors from the TME and exposure to chemotherapeutic agents are strongly linked to the development of MDR in cancer cells. Therefore, there remains a vast area for potential research to further elicit the interplay between factors existing both within and outside the TME. Elucidating the mechanisms within this network is essential for developing new therapeutic strategies that are less prone to failure due to the development of resistance in cancer cells.

## 1. Introduction

Tumour cells exist within a tumour microenvironment (TME) comprising signalling molecules and the stroma, which includes vasculature, immune cells, fibroblasts, and the extracellular matrix (ECM) [1,2,3,4,5]. The TME can be distinguished from the environment of non-cancerous cells by several factors, particularly a fluctuation in oxygen and nutrient availability, a low pH, and an excess of free radicals [6,7,8,9]. Adaptation to this characteristic environment has been shown to foster cell survival and proliferation, thereby promoting the transformation of cells into a malignant phenotype [6,9,10,11,12]. Additionally, many of these TME factors promote cancer development and metastasis. Indeed, it has been reported that the TME increases the mutation frequency within tumours, notably in critical genes such as *TP53*, causing genetic instability that is known to contribute to tumour progression [13,14,15,16]. Therefore, the TME can be viewed as a “cancer-prone environment”.

The development of this cancer-prone microenvironment has been strongly linked to the exposure of cells to environmental stress [17,18]. A notable and well-studied effect of environmental stress is the induction of inflammatory mediators and the production of reactive oxygen species (ROS), which are important drivers of oncogenesis, angiogenesis, metastasis, and the development of multidrug resistance (MDR) [17]. The higher levels of ROS found in cancer cells have been linked to the activation of numerous transcription factors, of which nuclear factor kappa-light-chain-enhancer of activated B cells (NF-κB) and hypoxia-inducible factor 1-alpha (HIF-1α) are some of the most important [17,19,20,21,22,23,24,25]. These transcription factors collectively alter the expression of hundreds of genes involved in tumourigenesis and regulate the expression of immune checkpoints (CTLA-4, PD-L1), cell-cycle regulatory molecules (cyclin D1, TGF-α) and important genes required for cell transformation, proliferation, and metastasis (PTEN, VEGF, HSP 90) [22,23,26]. Apart from external stimuli that cause oxidative stress, there are many cellular sources of ROS, including those produced due to changes in intracellular metabolic activity, mitochondrial activity, and increased oxidase activity [27]. Other factors present within the TME that contribute to chronic oxidative stress include the cells of tumour stroma, such as tumour-associated macrophages (TAMs) or myeloid-derived suppressor cells (MDSCs), which also produce inflammatory mediators and ROS [24,28,29,30,31]. However, these stressors will not be discussed in detail here. For a comprehensive review on TME-associated immune cells, see Labani-Motlagh et al., 2020 [32].

## 2. External Stress Mediates the Development of a Cancer-Prone Microenvironment

External stresses have been reported to mediate essential tumorigenic changes to the TME, most notably through the generation of ROS. These ROS include the superoxide anion (O_2_^•−^), hydrogen peroxide (H_2_O_2_) and hydroxyl radicals (OH^·^). ROS then react with and damage cellular lipids, proteins, and DNA, but they also serve as signalling molecules for essential biological processes [33,34,35,36,37]. This may have evolved as a mechanism for cell survival during environmental stress.

External factors leading to ROS production include (i) ultra-violet light [38] and ionising radiation, e.g., X-rays [39]; (ii) xenobiotics [40] and chemotherapeutics, most notably anthracyclines, alkylating and platinum agents [41]; (iii) bacterial infections, e.g., *Helicobacter pylori* [42]; (iv) viral infections, particularly, hepatitis viruses, human immunodeficiency virus, influenza A and Epstein-Barr virus [42]; (v) autoimmune disorders, such as vitiligo or irritable bowel syndrome [43,44]; (vi) allergens [45]; (vii) tobacco use and alcohol consumption [46,47]; (viii) obesity or a high-calorie diet [48]. Collectively, these external insults have been shown to elevate the amount of ROS within the TME either directly or via induction of an inflammatory response.

The relationship between inflammation and oxidative stress is well established [48,49,50]. Activated inflammatory cells, such as macrophages or leukocytes, are recruited to the site of damage, and due to their NADPH oxidase (NOX) activity, these cells can produce and release significant amounts of ROS, which contribute to the oxidative stress within the microenvironment [50,51,52]. They also produce soluble mediators, e.g., cytokines, chemokines, or metabolites of arachidonic acid, that drive further recruitment of inflammatory cells to the damaged site and increase the production of ROS – a vicious circle leading to chronic inflammation [48,52]. Most notably, it has been recently demonstrated that increased ROS production by myeloid cells can induce genome-wide DNA mutations in healthy neighbouring cells, which is sufficient to initiate tumour growth and promote tumour progression even in the absence of a carcinogen [53].

**Figure 1:**
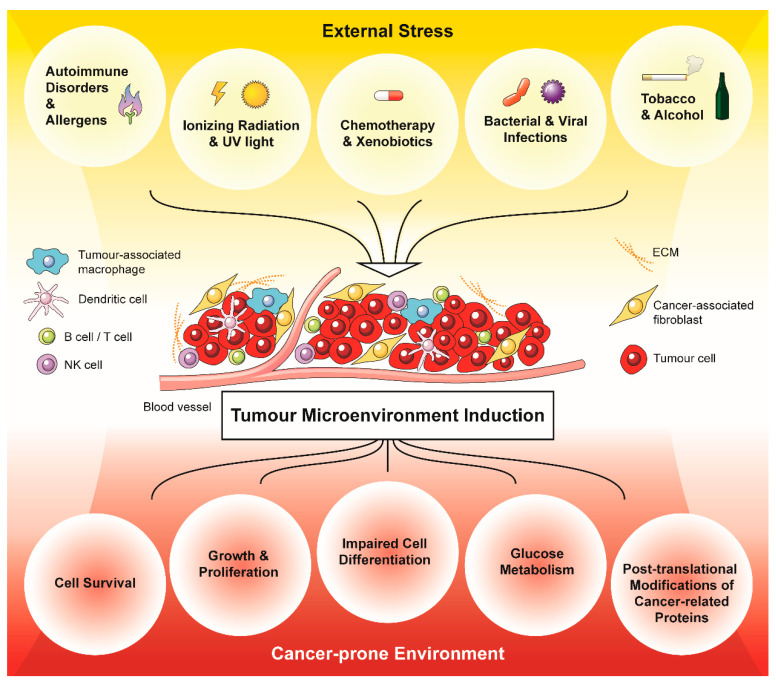
External stressors promote and drive a cancer-prone environment via the generation of oxidative stress in a tumour microenvironment (TME). Acute and chronic stressors generate oxidative stress in the form of ROS within the TME, which affects the composition of the tumour-associated stroma. In turn, the stress-induced tumour-associated stroma promotes cancer cell survival, growth and proliferation, impaired cell differentiation, glucose metabolism and post-translational modifications of cancer-related proteins.

Chronic inflammatory stimuli and consequent oxidative stress can cause direct effects such as gene mutations and post-translational modifications of key cancer-related proteins. Further, they may alter cell signalling pathways such as those involved in cell growth/proliferation, differentiation, protein synthesis, glucose metabolism, cell survival and inflammation [16,54,55]. Therefore, sustained environmental stress is strongly linked with cancer development and creates a cancer-prone niche essential for the survival of transformed cells, tumour proliferation, angiogenesis, and invasion (Figure 1). However, it is important to note that the ultimate effect of these ROS is complex and depends on their local concentration, the microenvironment, and the genetic background of the impacted individual [48].

## 3. Microenvironmental Stress and the Development of Drug Resistance

Drug resistance can be innate, arising prior to drug treatment, or acquired, developing in response to pharmacological insult [56,57]. This resistance to chemotherapeutic agents may be independent of their structure and pharmacological mechanism, known as MDR [57]. While the role of TME stress is crucial in malignant transformation and cancer progression, its involvement in the development of therapeutic resistance is a matter of current research [58]. The composition and organization of TME influence tumour heterogeneity and facilitate the selection of resistant clones [59], thus affecting cancer cell survival and therapeutic response to conventional cancer therapies [60].

**Figure 2:**
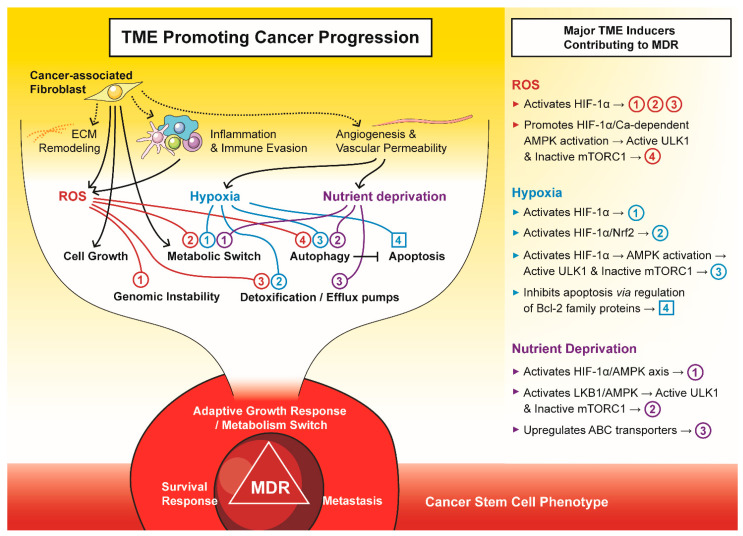
The tumour microenvironment (TME) promotes cancer progression to a multi-drug resistant (MDR) phenotype. The major components of TME, i.e., cancer-associated fibroblasts, reactive oxygen species (ROS), hypoxia, and nutrient deprivation, drive tumours to an adaptive growth, survival and metastatic phenotype that is often attributed to cancer stem cells.

Herein, we will discuss some of the major mechanisms involved in TME-mediated development of drug resistance, which include (i) increased survival and altered drug delivery via metabolic reprogramming; (ii) changes to stromal cells, including ECM remodelling; (iii) autophagy and insensitivity to apoptosis and (iv) the induction of a cancer stem cell (CSC) phenotype (Figure 2). Of course, the MDR-promoting elements of the TME are not limited only to these, but also include a number of other factors, such as the surrounding vasculature, which impacts the distribution of oxygen, nutrients and drugs, the ECM, which affects cell adhesion-mediated drug resistance [61], immune suppression mechanisms [62], or exosome-mediated trapping of therapeutic antibodies [63,64].

### 3.1. Metabolic Reprogramming, the ROS/HIF-Axis and the Development of Multi-Drug Resistance

The fundamental metabolic processes of cancer cells remain similar to those of cells in healthy tissues. However, cancer cell metabolism can be altered due to mutations or variations in the expression levels of genes encoding metabolic enzymes or the expression of alternative enzyme isoforms [6]. Malignant cells often display accelerated metabolism and high glucose and glutamine requirements and uptake [65,66,67]. In fact, these factors link the rewiring of cancer cell metabolism with stressors present within the TME, orchestrating tumour progression and resistance to therapy [60].

As a result of a rapid tumour expansion and limited diffusion from the local vasculature, proliferating tumour cells surpass the supply of oxygen and nutrients [68,69,70]. Studies have reported that the presence of nutrient and oxygen starvation in the TME initiates malignant transformation, tumour progression, angiogenesis, and metastasis and affects therapy response via mediation of the ROS/HIF-1α-axis [19,20,23,71]. Under starvation conditions, the process of HIF-1α hydroxylation by oxygen-dependent prolyl hydroxylases (PHD) is halted, which prevents tagging HIF-1α for subsequent ubiquitination and destruction [72,73]. This allows HIF-1α to accumulate and dimerise with a HIF-1β subunit that can bind to hypoxia response elements (HREs) in the nucleus [71]. Several models of oxygen sensing have been proposed [74]. Apart from direct inhibition of PHDs, ROS have been implied in post-translational modifications of the HIF-1α protein, activating the ERK and PI3/AKT pathways, or regulating microRNAs miR-21 and miR-210, all of which stabilise HIF-1α [74,75,76,77].

Furthermore, oxygen is required for the final transfer of electrons in the mitochondrial respiratory chain. During oxygen depletion, electrons accumulate in the mitochondrial respiratory compartments and reduce the existing O_2_ molecules to radicals, thus up-regulating ROS generation [71]. Indeed, hypoxia-induced ROS were reported in cultured cells [78,79] as well as animal models [80,81]. This phenomenon was then attenuated through the administration of antioxidants or inhibition of cellular mitochondrial respiration [79,80], underlining the role of mitochondria in response to hypoxic insult and regulation of HIF-1α [82].

The activation of HREs regulates the expression of many genes involved in a plethora of cellular processes, including those affecting the metabolism of cells. HIF-1α actively participates in metabolic adaptation of cancer cells to hypoxia by up-regulating the expression of genes encoding pyruvate dehydrogenase kinase 1 (*PDK1*), which inhibits the conversion of pyruvate into acetyl-CoA, glucose transporters (*GLUT1* and *GLUT3*) and carbonic anhydrase IX (*CA-IX*) converting the metabolically generated CO_2_ into carbonic acid [83,84]. These factors steer the cells from oxidative phosphorylation by the tricarboxylic acid (TCA) cycle towards glycolysis [85]. Indeed, hypoxic malignant cells exhibit a metabolic switch toward “Warburg” biology. In a process termed aerobic glycolysis, energy is created by metabolising glucose in a non-oxidative manner despite oxygen being available [86]. Although less efficient in producing ATP, aerobic glycolysis is preferentially used over mitochondrial oxidative phosphorylation in many cancer types [86], most likely because the side products of such metabolic mode are required for biomass production [6]. However, integral to cellular metabolic processes is the production of toxic by-products, including ROS, which are generated largely through the changes to mitochondrial metabolism [82]. Further, many antineoplastic compounds, including anthracyclines, alkylating or platinum agents, have been shown to produce oxidative stress that interferes with therapy and facilitates MDR development [41].

Consequently, the intracellular concentration of lactic acid, the end-product of glycolysis, is increased and needs to be extruded. HIF-1α is implicated in modulating the intracellular pH via regulation of the monocarboxylate transporter 4 (MCT4), a member of the H^+^/lactate co-transporter family that excretes lactate from cells [87]. Although the intracellular pH is maintained at an appropriate level allowing survival and proliferation, the TME becomes acidic. While tumour cells are well adapted to such conditions (e.g., via increased antioxidant protein expression) [88,89,90], H^+^ ions flowing to adjacent non-cancerous tissue create a toxic environment that induces apoptosis or necrosis in normal cells [91]. Low pH of the TME (pH 6.5–6.9) also promotes degradation of ECM via matrix metalloproteinases (MMPs) and cathepsins, increases angiogenesis through the release of VEGF and inhibits the tumour antigen-induced immune response, all of which facilitate local invasion, subsequent tumour growth and metastasis [91].

Many HIF-1α targets are pro-angiogenic factors, such as angiopoietin or VEGF [92,93]. Although these factors trigger the formation of blood vessels in hypoxic parts of the tumour bed [92,93], the tumour-associated vasculature is often poorly organised and inefficient. Therefore, diverse oxygen levels in the TME drive the heterogeneity of the tumour, creating populations of glycolytic and oxidative tumour cells [88]. Interestingly, lactate has been proposed to link glycolytic and oxidative metabolism in tumours in a “symbiotic” fashion [94]. Lactate flux has essential roles in adjusting intracellular acid-base balance [95]. It is also shuttled from hypoxic regions to oxygenated sites, where it is taken up via monocarboxylate transporter 1 (MCT1) by the oxidative tumour cell subpopulations to “fuel” their growth [94]. In fact, oxidation of lactate under aerobic conditions is known to be more concise and effective, leading to a preferential utilisation of lactate for fuelling the TCA cycle and sparing glucose for the highly glycolytic tumour cells in anaerobic tumour compartments [94,96]. Lactate can also act as a hypoxia mimetic factor by activating HIF-1α expression in normoxic cancer cells and adjacent endothelial cells [94]. A similar symbiotic relationship was also described between tumour cells and the TME stroma. ROS-producing oxidative cancer cells trigger a switch towards glycolysis in nearby fibroblasts, leading to the production of lactate, pyruvate and ketone bodies [88,97,98]. Such metabolic reprogramming of the surrounding stroma has been termed the “reverse Warburg effect” [99]. Products of glycolysis from both glycolytic cancer cells and fibroblasts are exported via MCT4, providing oxidative cancer cells with mitochondrial fuel [100]. Such crosstalk further promotes cancer progression and resistance; thus, disrupting this metabolic symbiosis presents a strategy for sensitising resistant tumours to anti-cancer therapy. In fact, some of the MCT1 inhibitors, such as SR13800 and AZD3965, have already entered Phase I and II clinical trials [96].

This evidence indicates that the complex metabolic rewiring establishes a nurturing niche that drives the tumour’s aggressiveness, which also correlates with poor prognosis in patients [88]. In particular, poor drug permeability in hypoxic and acidic TME and activated ROS/HIF-axis directly aid in developing and propagating MDR clones [17] (Figure 2). Anti-cancer compounds that are weak bases, such as doxorubicin, can be protonated in low extracellular pH, which leads to ion-trapping, reduced drug uptake and consequent MDR [101]. However, an acidic environment increased the resistance of cancer cells even to paclitaxel, a neutral drug not affected by pH [101]. Restoring neutral pH growth conditions in cultured cell lines or using metabolic modulators to inhibit glycolysis and glucose uptake results in a switch to oxidative phosphorylation, enhancing the toxicity of paclitaxel and doxorubicin [101]. The importance of glycolytic metabolism in conferring MDR is evident [102,103]. For example, resistance to doxorubicin observed in acute myeloid leukaemia cells was attributed to heightened expression of HIF-1α and increased glucose consumption [104]. Similarly, worse overall and disease-free survival of lung cancer patients was associated with high levels of HIF-1α, glucose transporter I and CA-IX detected in tumour tissues [105].

Tamoxifen resistance in breast carcinomas, which occurs in more than 40% of patients, was attributed to ROS and oxidative stress [88]. ROS induced by hypoxia mediates HIF-1α stabilisation, leading to activation of HIF-1α targets, such as VEGF-A [71,82]. Secretion of VEGF-A facilitated by the ROS/HIF-1α axis was shown to cause resistance to etoposide and doxorubicin [106,107], presumably via mechanisms related to improved vascular stability of the tumour [71]. In a melanoma cell line, VEGF-A expression stimulated the continued generation of ROS, further HIF-1α stabilisation and VEGF-A expression in an autocrine manner, thus resulting in continual etoposide resistance [106]. It was also shown that doxorubicin could up-regulate HIF-1α expression via nitric oxide synthesis, increasing VEGF secretion [107].

The redox-sensitive transcription factor HIF-1α has been demonstrated to up-regulate the expression of several ATP-binding cassette (ABC) transporters as well [48,108]. These pumps extrude various cytotoxic agents from cancer cells, reducing drug accumulation inside the tumour [109]. During TME stress, up-regulation of P-glycoprotein (Pgp) [17], the most consistently overexpressed ABC transporter involved in MDR [110], as well as other efflux pumps, including the multidrug resistance protein 1 (MRP1) [111] and 4 (MRP4) [112] or the breast cancer resistance protein (BRCP, also known as ABCG2) [113], has been induced directly by the ROS/HIF-lα axis. Furthermore, activation of the Tie2 receptor tyrosine kinase, a downstream target of HIF-lα [92], was attributed to MRP2 elevation and consequent resistance to cisplatin in malignant glioma cells [114]. Interestingly, recent findings have demonstrated that Pgp is endocytosed along with the plasma membrane and exists on lysosomal membranes [115]. There, it mediates the sequestration of drugs, e.g., doxorubicin, into lysosomes. As a result of acidic pH-mediated protonation, the drugs are trapped in the lysosomal lumen, unable to interact with their cellular targets [115]. The lysosomal mechanism of drug trapping is further enhanced by the greater acidity of lysosomes in MDR cells compared with drug-sensitive cells [116]. Therefore, the stress-inducing conditions of the TME may play a crucial role in inducing MDR not only via enhanced drug efflux but also by lysosomal sequestration [17,117] (Figure 2).

In response to oxygen deprivation, many genes, including those encoding microRNAs, are modified and deregulated. While some of these miRNAs are induced by HIFs [76,118], others can affect the expression of HIFs and modulate the HIF-1α response pathway [119,120,121]. In addition, up-regulated miR-98 under hypoxia potentiated resistance to cisplatin and doxorubicin in head and neck squamous carcinoma cells [122].

Naturally, signalling in hypoxia is not limited only to the HIF-1α axis. For example, increased ROS also promote the nuclear localisation of NF-κB, which enhances transcription of the *HIF1A* gene and its downstream targets [123]. Nuclear factor (erythroid-derived 2)-like 2 (Nrf2) controls expression of antioxidant-response genes, thus regulating ROS and maintaining oxidative homeostasis [124] (Figure 2). However, recent evidence points at a pro-carcinogenic role of Nrf2 via activating and sustaining the HIF-1 response [37]. Nrf2, by signalling through thioredoxin, was shown to elevate levels of HIF-1α [125]. On the other hand, HIF-1α can decrease the thioredoxin reductase level, potentiating the Nrf2 signal [126]. Furthermore, high levels of MDR-related ABC transporters, including Pgp [127], MRP1 [128] or ABCF2 [129], have been associated with Nrf2 expression and resistance to multiple drugs. In fact, both HIF-1α and Nrf2 stress response pathways exist in a complex, interactive signalling network that stimulates tumour progression, angiogenesis, metabolic shifts and chemoresistance [102]. Therefore, in the context of hypoxic TME, targeting only one MDR-promoting pathway might not be a good therapeutic approach.

### 3.2. Stromal Cells and the TME

Along with the pathologically altered parenchyma, the tumour cells, TME also consists of stroma, including (i) non-malignant cells, such as fibroblasts, specialised mesenchymal cell types, immune cells, and vasculature with endothelial cells and pericytes, and (ii) components of ECM and signalling molecules [130,131]. This holds true not only for solid tumours but also for hematopoietic malignancies where secondary lymphoid organs or bone marrow serve as TME sites [132]. Physiologically, the stroma is essential for maintenance and integrity in normal tissues, thus sustaining the homeostasis of tissues. However, changes in the stroma can cause dramatic alterations in the whole system, hence creating a cancer-favouring microenvironment [130]. Furthermore, the stromal elements of the TME are not simple bystanders but exhibit diverse and often divergent effects in tumorigenesis and anti-cancer therapy. While some of the immune cells (e.g., CD8^+^ T cells or NK cells) possess tumour-suppressing activities [32], other stromal cells of the TME have been implicated in promoting cancer progression, metastasis and MDR [5,130]. Among these, cancer-associated fibroblasts (CAFs) are most likely the best-studied cell type of the TME stroma.

To better understand the relationship between tumour cells and stroma within the TME, a “Tumour–Stroma Co-Evolution” model has been proposed [133]. Most of the activated CAFs are transformed from resident fibroblasts after stimulation by fibroblast growth factor (FGF), monocyte chemotactic protein 1 (MCP-1), platelet-derived growth factor (PDGF), tissue inhibitor of metalloproteinase 1 (TIMP-1) or tumour transforming growth factor β (TGF-β) present in the microenvironment [64]. CAFs may also arise from bone marrow-derived mesenchymal stem cells, epithelial or endothelial cells within the TME [134]. Cancer cells induce oxidative stress in the adjacent stroma, which mimics the effects of hypoxia even under aerobic conditions. This results in down-regulation of Caveolin-1 (Cav-1) and activation of HIF-1α and NF-κB response pathways that collectively drive metabolic reprogramming, ROS production and confer the CAF phenotype [88,133]. Consequently, CAFs become proliferative, migratory and highly secretory cells, thus supporting tumour progression and allowing cancer cells to evade therapy. In addition, chemotherapy-induced DNA damage in the TME can promote the development of a CAF phenotype in fibroblasts, creating a highly glycolytic and pro-inflammatory niche that subsequently activates autophagy and stemness in nearby cancer cells [64] (Figure 2). Indeed, increased numbers of CAFs and genetic changes in the tumour-associated stroma, including loss of Cav-1 or enhanced MCT4 expression [88], were linked with a poor clinical prognosis in several cancers [135,136,137,138,139].

In turn, activated CAFs produce ROS, promoting genomic instability in tumour cells and driving their evolution towards a more aggressive and resistant phenotype [64,140,141,142]. In fact, CAF-induced oxidative stress was sufficient to induce breast cancer tumour growth [143]. Enhanced cytokine synthesis and secretion also impair drug sensitivity in adjacent tumour cells, triggering soluble factor-mediated drug resistance. Such a secretome includes FGF7, PDGF, VEGF, hepatocyte growth factor (HGF), stromal cell-derived factor 1 (SDF-1) or interleukin 6 (IL-6) [64]. CAFs also participate in activating the Wnt/β-catenin signalling pathway in the nearby tumour cells [144]. In fact, increased Wnt signalling has been reported to induce therapeutic resistance in glioblastoma, ovarian cancer or non-small cell lung carcinoma [144], most likely by increasing the expression of ABC transporters, such as Pgp [145] or BCRP [146]. Such a secretory phenotype of CAFs is also induced after chemotherapy. For example, docetaxel and mitoxantrone treatment led to secretion of WNT16 in CAFs and promoted Wnt signalling [147] while doxorubicin-induced the production of IL-6 and TIMP1 [148].

Resistance to targeted therapy can also be acquired via CAFs [130]. Anti-angiogenic treatment using bevacizumab led to up-regulation of VEGF-A and FGF2 in stromal cells in a mouse model of lung cancer [149]. After such treatment in myeloma tumours, CAFs were able to reactivate angiogenesis through PDGF-C signalling [150]. CAF-secreted growth factors, e.g., EGF, FGF and HGF, render resistance of cancer cells to multiple tyrosine kinase inhibitors (TKIs) [151,152,153]. These growth factors activate proliferative signalling by binding to their respective receptors, most notably through PI3K-AKT or mitogen-activated protein kinase (MAPK) pathways. Moreover, cross-activation of signalling pathways downstream of the activated receptors can also facilitate resistance to the TKIs [144].

ECM components secreted by CAFs are different to those produced by non-transformed fibroblasts [130]. Apart from abnormal collagen secretion, the tumour ECM contains tenascin or periostin, is more stiff and contractile, has altered organisation [130], and is also able to downregulate the expression of the tumour suppressor PTEN in cancer cells [154]. Furthermore, dense ECM of the TME reduces the concentration of anti-cancer agents in several ways: (i) Rigid ECM can reduce blood vessel density and creates a physical barrier through which therapeutics cannot diffuse [155,156,157]. (ii) Higher interstitial pressure of dense ECM prevents agents from entering the tumour mass [158]. (iii) CAFs express cytochrome P450s (CYPs) [159,160] that metabolise a variety of drugs, e.g., docetaxel metabolised by CYP3A4 [159,161]. In fact, particular CAF-derived molecules were reported to aid MDR development, including increased type I collagen or hyaluronan production [134]. CAFs also remodel the ECM to a greater extent, most notably by expressing MMPs [144]. This promotes plasticity and invasiveness of cancer cells and can result in chemoresistance [146].

CAFs can also produce exosomes which are lipid membranous vesicles filled with various factors and signalling molecules that can be internalised into cancer cells via endocytosis or phagocytosis [144]. These vesicles have been reported as another driving force of drug resistance. For example, Pgp present in CAF-derived exosomes increased drug efflux from cancer cells and activated pro-survival signalling [144]. Similarly, microRNA miR-21 transported by exosomes silenced apoptotic protease activating factor 1 (APAF1), thus causing resistance to paclitaxel in ovarian cancer cells [162].

The formation of blood vessels during malignant progression is a critical survival property of cancer cells acquired at an early stage of tumorigenesis [163]. Blood vessels consist of endothelial cells, which create a tight barrier between the blood and tissue and interact with ECM. Abnormal angiogenesis is a feature of tumour progression, where hyperproliferating cancer cells surpass their blood supply and become hypoxic. This hypoxic environment, via activation of HIF-1α and the VEGF pathway, creates an imbalance between the production of pro- and anti-angiogenic factors, leading to the rapid and disorganised formation of blood vessels [164,165]. Indeed, studies have shown that HIF-1α and VEGF overexpression are associated with cancer aggressiveness and poor overall survival of cancer patients [163,165,166,167,168,169]. Activating this “angiogenic switch” is essential for the adequate supply of nutrients and oxygen to the tumour, allowing excessive growth and metastatic spread by facilitating the extravasation, circulation and relocation of tumour cells [165]. These tumour blood vessels differ from normal vasculature in architecture. While normal vasculature has a highly organised architecture, the vasculature within a tumour is typically immature, with increased vascular permeability and turbulent blood flow [165,170].

Rapid cancer-cell proliferation and the presence of CAFs within host tissue generate physical forces that can be transmitted by the ECM. This produces a growth-induced solid stress, compressing blood vessels and contributing to impaired perfusion [171,172]. The resulting hypoxia and acidity in the tumour microenvironment contribute to disease progression [172,173,174,175]. The leakiness and compression of tumour vessels depend on the tumour type, stage, and location, varying within the same lesion and between lesions of the same patient [175]. These changes in the tumour microenvironment have also been linked to the development of drug resistance. Endothelial cells from highly metastatic tumours have been reported to express higher levels of pro-angiogenic genes and stemness genes, such as stem cell antigen-1 (*SCA1*), multidrug resistance 1 (*MDR1*), and aldehyde dehydrogenase (*ALDH*), which all contribute to the development of drug resistance [176,177,178,179].

Fifty years ago, anti-angiogenic therapy was first proposed as an anti-cancer therapy by Judah Folkman [180]. Since then, numerous agents have been developed that target tumour blood vessels either by inhibiting the formation of new capillaries or destroying existing tumour blood vessels [163]. The success of bevacizumab, a monoclonal antibody to VEGF, in metastatic colorectal cancers has led to the development of other anti-angiogenic therapies [163,181]. However, their success has been limited by the development of resistance following alternative mechanisms of blood vessel formation, and extended exposure has been linked to hypoxia-related tumour regrowth and an aggressive and metastatic phenotype [163,182,183].

**Figure 3:**
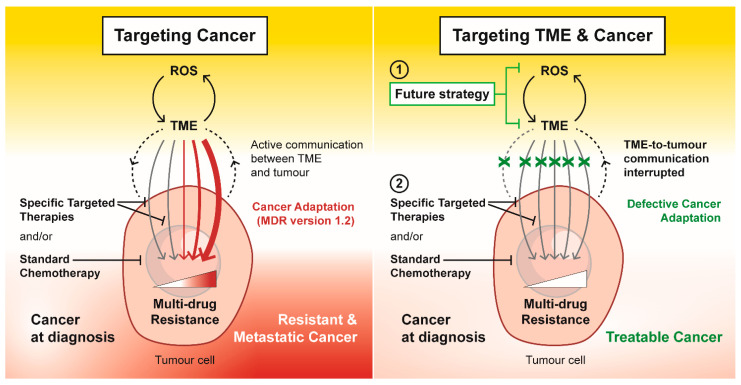
Targeting tumour microenvironment (TME) as a future strategy to overcome multi-drug resistance. In response to specific targeted therapies and standard chemotherapeutics, external and internal stress within the TME drives and promotes cancer adaptation in the form of drug resistance and metastasis. Targeting the ROS-TME circuit interrupts the TME-to-tumour communication that maintains a multi-drug resistance phenotype. This future strategy to target TME has the potential to disrupt the cancer adaption response, which could re-instate the efficacy of specific targeted therapies and standard chemotherapeutics in an attempt to treat cancer successfully.

Due to intrinsic resistant phenotypes within the cancer cell populations, unimodal anti-cancer treatments do not successfully eliminate all cancer cells. Moreover, most therapies spare the cancer-associated stroma, which assists in repopulating the TME with resistant cancer cells, resulting in cancer relapse and recurrence of more aggressive tumours. Considering this evidence, anti-cancer therapeutic strategies should be multimodal, and they should involve approaches that target and constrain the tumour stroma or revert the stroma to a tumour-suppressive state (see also Figure 3).

### 3.3. The TME Modulates Autophagy and Apoptosis to Enhance Cancer Cell Survival

In order to ensure homeostasis in tissues, cells possess an inherent mechanism of self-destruction, called apoptosis or programmed cell death [184]. Apoptosis normally occurs during development or ageing and presents a defence mechanism of eliminating damaged or defective cells. Two main pathways initiate apoptosis: (i) an extrinsic pathway activated by death ligands binding to corresponding death receptors and (ii) an intrinsic pathway that is triggered by an excess of pro-apoptotic to anti-apoptotic BCL-2 family proteins in mitochondria, which can be initiated by a number of stimuli, such as a lack of growth factors, hypoxia, hyperthermia, viral infections, ROS, toxins, or radiation [184]. Both pathways initiate an energy-dependent cascade of events that involves the activation of cysteine proteases called caspases. These further activate endonucleases and proteases that mediate the breakdown of cell molecules and lead to controlled cell death [184].

Chemo- or radiotherapy kills cancer cells primarily by inducing apoptosis. Therefore, resistance to cell death presents an essential feature of cancer development and tumour cell survival, resulting in therapy resistance [185]. In general, cancer cells exploit a variety of mechanisms to suppress apoptosis, including elevated expression of anti-apoptotic proteins, down-regulation or mutation of pro-apoptotic proteins, alteration in the p53 pathway or up-regulation of the PI3K/AKT axis [185]. The conditions and components found within the TME can influence these factors and subsequent sensitivity to cell death.

Hypoxia and increased HIF-1α signalling are significantly correlated with survival, decreased expression of pro-apoptotic factors and increased expression of anti-apoptotic factors [102] (Figure 2). For example, hypoxia-mediated resistance to etoposide observed in colon cancer cells was attributed to the decrease in the BCL-2 family of proteins, which promotes apoptosis by releasing cytochrome *c* from mitochondria, initiating caspase activation [186]. Reduced levels of BH3-interacting domain death agonist (BID) and BCL-2-associated X (BAX) proteins correlated with the degree of oxygen deprivation. In fact, BID expression was repressed by HIF-1 that binds to the *BID* promoter [186].

The apoptotic threshold in cancer cells is also elevated by CAFs, most notably by producing ROS and various soluble factors (Figure 2). ROS generation drives defence activities against oxidative stress in neighbouring cancer cells, particularly through inducing the expression of antioxidants (e.g., peroxiredoxin1) and anti-apoptotic proteins (e.g., TIGAR) [133]. Among soluble factors produced by CAFs, FGFs suppress the expression of pro-apoptotic BCL-xL and apoptosis-inducing factor (AIF) [144]. Likewise, VEGF is not only a mitogen but also a survival factor that modifies apoptotic signalling in the cells of the tumour and the surrounding vasculature [122]. Indeed, VEGF-facilitated expression of anti-apoptotic proteins, BCL-2, MCL-1 or XIAP, and activation of the PI3K/AKT survival pathway protected colorectal cancer, breast cancer or leukaemia cells from apoptosis [122]. In the case of multiple myeloma (MM), the MM cells make adjacent fibroblasts secrete IL-6 that, in return, protects the MM cells from apoptotic stimuli and chemotherapy by promoting JAK/STAT signalling and expression of BCL-xL [122]. Resistance to cytarabine, 5-fluorouracil or oxaliplatin in leukaemia cells was attributed to signalling conferred via the CAF-secreted SDF1 that binds to the chemokine (C-X-C motif) receptor 4 (CXCR4) on these cells, thus elevating transcriptional activation of c-MYC and BCL-xL [134]. In addition, apoptosis in chronic lymphocytic leukaemia (CLL) cells was prevented through Bcl-2-dependent pathways as a response to IL-4 [187], INFα [188] and bFGF factors [189]. Interestingly, CAFs were reported to induce the expression of DNA methyltransferase 1 (DNMT1) in a pancreatic carcinoma model, causing hypermethylation and subsequent epigenetic inhibition of STAT1 and reduced expression of caspases 3, 7, 8 and 9 [122].

Other tumour-associated stroma cells also secrete growth factors that enhance tumour development and help cancer cells evade death [122]. For instance, adipocytes present in the TME protected leukaemia cells from chemotherapy treatment by stimulating the expression of anti-apoptotic proteins BCL-2 and PIM2 [190].

One of the key pathways facilitating stress-induced metabolic adaptation and damage control is autophagy. Organelles, proteins, or portions of the cytoplasm are sequestered into vesicles called autophagosomes, and after fusion with acidic lysosomes, the sequestered contents are degraded. In this way, cells eliminate damaged or harmful components and recycle nutrients to maintain energy homeostasis and survive unfavourable conditions [191]. Autophagy is regulated by several autophagy-related genes (*ATGs*) and their products [192]. It frequently occurs during tumorigenesis or cancer treatment as a response to stress stimuli present in the TME, including nutrient depletion, hypoxia or redox stress (Figure 2).

Depending on the type of stress, autophagy can be triggered through different pathways (Figure 2). Notably, adenosine monophosphate-activated protein kinase (AMPK) integrates several stress stimuli with the initiation of autophagy. Generally, AMPK phosphorylation leads to activation of the unc-51-like autophagy activating kinase 1 (ULK1) and repression of the mechanistic target of rapamycin complex 1 (mTORC1), leading to autophagy induction [193,194,195].

During starvation, AMPK is activated (i) in response to changes in the energy status of the cell by monitoring its AMP: ATP ratio or by (ii) phosphorylation by several upstream kinases activated by energy depletion, e.g., liver kinase B1 (LKB1) [191]. Besides AMPK, starvation-induced autophagy is also stimulated by c-Jun N-terminal kinase 1 (JNK1), which phosphorylates BCL-2, reducing its affinity for the BH3 domain of beclin 1, important in the formation of the autophagosomal membrane. Furthermore, liberating BCL-2 from beclin 1 complexes leads to blocking the intrinsic pathway of apoptosis [196].

In the case of hypoxia or oxidative stress, AMPK is stimulated through metabolic changes and HIF-1α activation [191]. Apart from inducing the expression of essential autophagy genes, such as *ATG8/LC3* or *ATG5* [191], HIF-1α also activates the expression of BCL-2/adenovirus E1B 19-kDa interacting protein 3 (BNIP3) and the BNIP3 like (BNIP3L) protein, both members of the BH3-only subfamily of BCL-2 proteins [197]. They form heterodimers and antagonise the activity of pro-survival BCL-2 or BCL-xL. However, they also disrupt the BCL-2–beclin 1 interaction, inducing autophagy in hypoxia [191,197].

Indeed, TME stress-induced autophagy promotes cancer cell survival and catalyses the development of MDR [191]. Not surprisingly, anti-cancer treatment is among stress stimuli that trigger autophagy [198]. Autophagy-mediated MDR has been demonstrated after treatment with numerous drugs, including paclitaxel, tamoxifen, epirubicin or trastuzumab [199]. In leukaemia cells, adriamycin and vincristine up-regulated the expression of S100 calcium-binding protein A8 (S100A8), which is required to form beclin 1 complexes and autophagosome [200]. Similarly, miRNAs targeting *ATGs* are important modulators of MDR [198]. Cisplatin down-regulated the miR-199a-5p in hepatocellular carcinoma cells, which led to autophagy activation and resistance to this agent [201].

TME stress also triggers autophagy in the stromal compartment and is closely linked with metabolic reprogramming and the “reverse Warburg effect” described above [88]. As CAFs lose their mitochondria by enhanced autophagic degradation (mitophagy), their metabolism is steered towards aerobic glycolysis [133]. Overproduction of recycled nutrients (products of autophagy), pyruvate, lactate or ketone bodies (products of glycolysis) and their efflux from CAFs collectively fuel the anabolic growth of cancer cells and drive cancer aggressiveness [133]. Similarly, adjacent cancer cells can use the fatty acids released from adipocytes due to autophagy to proliferate [88].

### 3.4. TME Induces a Cancer Stem Cell (CSC) Phenotype

The CSC model posits that the growth of a tumour is driven by a specific population of tumour cells with stem cell-like characteristics [202]. These CSCs have three distinguishing criteria, i.e., they are self-renewing, tumour propagating and can differentiate into all other cancer cells within the respective malignancy [203]. CSCs have been reported in many cancer types, including sarcoma, leukaemia, breast, colorectal and brain cancers [202,204,205]. These cells are reported to arise from the dedifferentiation of non-propagating tumour cell subsets [206]. Indeed, the stem cell-like features are important for cancer progression, as undifferentiated primary tumours are more likely to result in distant metastasis and a poor response to therapy [207].

The classical viewpoint assumed that tumour cells follow a hierarchy, whereby the tumour is fuelled by the long-term and slowly proliferating CSCs while largely being composed of non-CSCs that are only capable of transient proliferation [206]. However, a newer model suggests that CSCs are not necessarily rare or quiescent and that they can also arise by dedifferentiation and reprogramming of non-CSCs [206]. Similar to normal stem cells, CSCs may undergo symmetric-cell division to self-propagate; asymmetric-cell division to produce more differentiated progeny and self-renew; or they may become quiescent, depending on the stimuli the cell receive [208,209,210,211]. However, within the context of dysregulated signalling and genetic/epigenetic aberrations, the same processes that tightly control embryonic development, tissue regeneration, or wound healing are derailed in cancer. Consequently, CSCs experience a continuous expansion and production of more differentiated non-CSC progeny.

It has been reported that highly aggressive cancers hijack transcription programs of embryonic development and attain a more dedifferentiated stem cell-like phenotype, as measured by a stemness index derived from transcriptomic and epigenetic data of pluripotent stem cells and their derivatives [212]. This stem cell-like state was linked to the expression of transcription factors known to drive pluripotency, such as SOX2 or OCT4 [212]. This large-scale analysis also suggested a link between the immune microenvironment and cancer stemness for many tumours, i.e., higher stemness indices were associated with a lower leukocyte infiltration and lower programmed death-ligand 1 (PD-L1) expression [212]. Therefore, it would be expected that tumours with enhanced stemness would be less susceptible to immune checkpoint blockade treatments.

Depending on the TME, the population of CSCs demonstrates a dynamic quality, whereby CSCs may maintain, gain or lose the stem-like phenotype, resulting in heterogeneous populations of tumour cells with the potential to rapidly grow [206]. Stress within the TME has also been strongly linked to the development and maintenance of CSCs [213]. Stress-induced reprogramming is a new concept whereby stress in the TME, e.g., from hypoxia or chemotherapy, can activate reprogramming cascades that result in the dedifferentiation of tumour cells to a more stem-like state with the ability to maintain or reconstitute the malignancy (Figure 2) [206,214]. A variety of cancer types, including glioma, lung cancer and hepatoma cancers, have been reported to undergo stress-induced reprogramming [214]. Interestingly, hypoxia has also been found to dedifferentiate cells derived from normal human embryonic stem cells back into a stem cell-like state [215]. In addition to this, hypoxia, by increased cellular ROS signalling, has been demonstrated to activate AMPK through a calcium-dependent pathway [216]. Activated AMPK promotes tumour cell survival by increasing mitochondrial fatty acid oxidation, mitophagy-mitochondrial fission and mitochondrial biosynthesis [205]. Indeed, CSCs have been reported to be maintained in their stem-like state by AMPK activation although contradictory results on the role of AMPK in CSCs have also been published [217,218]. Nevertheless, in already developed tumours, AMPK appears to act as a tumour promoter, most likely by enhancing the survival of tumour cells under stress conditions [219]. Therefore, hypoxia in the TME may promote cancer-cell progression and a drug-resistant phenotype by coordinating induction and selection of the CSC tumour cells [220]. As a consequence of this, approaches that target the hypoxic TME in combination with standard chemotherapy may provide a promising strategy for eradicating CSCs.

Cell plasticity, in particular, the ability of CSCs to adopt a quiescent state, has also emerged as an important driver of drug resistance. Several studies have provided evidence that CSCs can undergo phenotypic transitions in response to appropriate stimuli from TME [202,213,221,222,223]. Drug resistance mechanisms exploited by CSCs include resistance to redox stress, the ability to repair damaged DNA, and an enhanced capacity to efflux anti-cancer drugs through ABC transporters such as ABCG2 [224]. Through these mechanisms, CSCs can efficiently evade chemotherapy, which explains why many patients relapse after treatment [202]. Moreover, as suggested by genetic-fate mapping, it is most likely the quiescent CSCs that form the residual population of chemotherapy-resistant tumour cells responsible for tumour re-growth and disease recurrence [202,225,226,227,228]. Understanding the mechanisms of how TME contributes to the regulation of CSC dormancy is of great importance for developing therapeutic interventions that would prevent the switching of CSCs to the highly resistant quiescent state.

## 4. Clinical Use of Agents Targeting the Stress Factors within the TME

Most available anti-cancer therapies are aimed mainly at the tumour cells, targeting their rapid growth or specific characteristics while omitting other tumour-promoting factors present within the TME. Although such an approach eradicates a significant part of the tumour mass, it often induces the selection of more resistant clones of cancer cells, inevitably leading to recurring refractory tumours and metastasis. Therefore, using agents targeting cancer cells and the cancer-prone environment is crucial for efficient and successful anti-cancer treatment (Figure 3). Different approaches and specific drugs that are discussed below have already been investigated in cancer clinical trials targeting (i) the ROS/HIF axis, (ii) stroma cells, (iii) apoptosis or autophagy, and (iv) CSCs.

### 4.1. Targeting the ROS/HIF Axis

While basal levels of ROS are required for a number of processes maintaining cell homeostasis, increased ROS production due to external stimuli, activation of oncogenes, hypoxia, or other stressors in the TME is inherent to tumours, making ROS a tempting therapeutic target [229]. However, the function of ROS in cancer cells is more complex than first envisioned. Current theories suggest that modestly elevated ROS are oncogenic and may confer a survival advantage. By contrast, ROS production, which is often increased during chemo- or radiotherapy, can reach a critical threshold that leads to cell death, thus serving as a tumour suppressor [229].

There are two divergent approaches to ROS-modulating therapies (Table 1). The antioxidant approach aims at scavenging ROS in cancer cells, thus inhibiting pro-survival signalling [230]. This approach includes dietary and supplementary antioxidants [231,232,233,234,235], glutathione (GSH)-inducing phytochemicals [236,237], NADPH oxidase inhibitors [238] or modifying cyclic nitroxides, which present a group of stable radicals with strong antioxidant properties [239]. Conversely, a pro-oxidant approach boosts ROS to cytotoxic levels, overcoming antioxidant systems and inducing cancer cell death [230,240]. This can be achieved by using inhibitors of the antioxidant systems [241,242,243,244,245,246,247,248] or by using exogenous stimuli that cause oxidative stress, e.g., radiotherapy or most conventional chemotherapeutics [249,250,251]. Interestingly, even molecular targeted therapies, including tyrosine kinase inhibitors and monoclonal antibodies (Table 1), exhibit ROS-mediated anti-cancer effects [252,253,254,255]. However, both pro- and anti-oxidant approaches cannot be used universally for all malignancies as they should ideally shift the redox status over the threshold in every tumour cell to be effective against the respective tumour. This might be a particular issue for ROS-inducing agents with a narrow therapeutic window. ROS depletion is, therefore, more suitable for tumours with modest ROS levels while increasing oxidative stress for tumours with higher levels of ROS [240]. Of course, the right choice for either of the strategies should depend not only on the tumour’s redox status but also on the activation of the redox-sensitive transcription factors, such as HIF, AP-1 or NF-κB [256].

In this regard, efforts have been made to develop anti-cancer therapeutics specifically targeting the HIF-1α regulation pathway, which is crucial for the survival of tumour cells. Multiple methods of targeting HIF-1α have been explored, including inhibition of HIF-1α (i) mRNA expression [257], (ii) protein synthesis [258,259,260,261,262,263,264,265,266], (iii) stabilisation [267,268,269,270], (iv) dimerization [271], (v) DNA binding [272], (vi) transcriptional activity [273], (vii) inhibition of HIF-1α at multiple levels [274,275], or (viii) HIF-1α degradation [276]. Table 2 shows an example of molecules interfering with the HIF-1α pathway that have been explored in clinical trials. Additionally, there are currently several ongoing trials of HIF inhibitors in cancer (NCT03216499, NCT03108066, NCT02293980, NCT03401788, NCT03634540, NCT02212639, NCT01652079). It is important to note that most of the inhibitors developed so far are not specific for HIF-1α but work indirectly by inhibiting other pathway components. Nevertheless, HIF-1α remains a viable therapeutic target for modulation, given its key role in tumour growth, invasion and drug resistance.

### 4.2. Stroma-Targeting Therapies

The increasing understanding of the importance of stroma in tumour progression has led to the development of several stroma-targeting strategies that have been investigated in clinical trials (Table 3) [130].

Using agents targeting the ECM reduces the density of its components and improves the diffusion of therapeutics [130,280,281,282,283,284,285,286]. Apart from degrading the ECM, other approaches focus on more efficient penetration of agents through stiff tumour stroma, e.g., by conjugation with albumin [287]. Targeting proteins expressed specifically by stromal cells can be used to modulate their proliferation, cytokine secretion and ECM formation [288,289,290]. Similarly, inhibition of metabolising enzymes in stromal cells, e.g., CYP3A4, can enhance the cytotoxic activity of used drugs [159]. Cancer cell receptors for factors secreted by stromal cells may be targeted to directly inhibit the tumour mass and disrupt the cancer cell-stroma signalling interactions [134,291,292,293,294,295,296,297]. Although still in preclinical studies, cancer vaccines specific for stromal antigens hold great potential for future therapies [130,298,299]. For example, using chimeric antigen receptor T (CAR-T) cells reprogrammed to recognise fibroblast-associated protein (FAP) specific for CAFs stimulated the anti-tumour immunity and caused tumour regression even without the addition of any cytotoxic agent. Of course, when combined with other drugs, these vaccines can make the strategy even more effective [130]. Since inflammation is known to mediate the development of cancer-prone microenvironment and promote cancer progression [48], pro-inflammatory immune cells in the TME have become a novel target in anti-cancer therapies (Table 3) [300,301]. Other drugs, e.g., non-steroid anti-inflammatory drugs (NSAIDs) or corticosteroids (Table 3), have been suggested in cancer prevention [302,303,304,305]. Their anti-inflammatory properties also neutralise the cancer-promoting stromal cells and hence could be employed in anti-cancer combined therapy [303]. In addition, a well-established link between inflammation and ROS [48] further highlights the role of antioxidant strategies in anti-cancer treatment (Table 1).

**Table 3:** Agents explored in clinical trials targeting the tumour stroma.

Stromal Targets	Compounds Involved in Cancer Clinical Trials
***ECM***	
collagen type I	nanoparticle albumin-bound paclitaxel [287], halofuginone [285]
hyaluronic acid	PEGPH20 [282]
integrins	cilengitide [281]
lysyl oxidase	all-trans retinoic acid (ATRA) [280], calcipotriol [284]
matrix metalloproteinases	marimastat [286]
***Stroma-specific proteins***	
CYP3A4	clarithromycin, itraconazole [159]
FAP	ATRA [289], sibrotuzumab [288], RO6874813 [290]
***Cancer cell-stroma signalling***	
CXCR4	plerixafor [296]
FAK	defactinib [291]
FGFR	AZD4547 [293], dovitinib [294]
TGFβ	fresolimumab, galunisertib [295]
VEGF	aflibercept, bevacizumab [306], PTK787 [297]
VEGFR	pazopanib, sorafenib, sunitinib, vandetanib [292]
***Inflammation inhibition***	
pro-inflammatory immune cells	gemcitabine [301], sunitinib [300]
mediators of inflammation	celecoxib [307], dexamethasone [304], metformin [302], NSAIDs [305]

CXCR4, CXC-chemokine receptor 4; CYP3A4, cytochrome P450 3A4; FAK, focal adhesion kinase; FAP, fibroblast-associated protein; FGFR; NSAIDs, non-steroid anti-inflammatory drugs; TGFβ, transforming growth factor β.

It is evident that strategies that target and constrain the tumour stroma might have curative outcomes, especially when the stroma facilitates tumour growth and resistance to therapy. On the contrary, when the stroma performs tumour suppressive functions, such approaches might have undesirable effects [130]. Importantly, targeting the tumour stroma alone will presumably not eliminate the entire tumour; thus, combinational strategies targeting both tumour mass and stroma are essential for favourable outcomes in patients.

### 4.3. Clinical Use of Autophagy and Apoptosis-Targeted Therapies

Cancer is a process when too little apoptosis occurs, resulting in tumour growth and MDR. Interestingly, cancer cells are often more sensitive to therapy-induced apoptosis than normal tissues, likely due to oncogenic stress or environmental stimuli such as hypoxia or insufficient nutrition [308]. Therefore, modulation of apoptotic threshold and exploiting the cell’s own mechanism for death present an attractive anti-cancer strategy.

Years of research have led to the development of various drugs that target different stages of both intrinsic and extrinsic apoptosis pathways. Generally, two approaches can be employed (Table 4): (i) stimulation of the pro-apoptotic molecules [205,309,310,311,312,313,314,315] or (ii) inhibition of the anti-apoptotic molecules [205,310,316,317,318,319,320,321,322,323]. Several of the investigated compounds (Table 4) are plant-derived (e.g., curcumin or quercetin), and apart from exerting pro-apoptotic effects on tumour cells, they present an effective means of cancer prevention or chemoprotection [309,320]. Moreover, many conventional treatments, including radio- and chemotherapy, induce apoptosis in cancer cells indirectly by the production of ROS [230,324]. For example, it has been shown that ROS stimulate the activity of caspases, up-regulate the death receptor 5 (DR5) or affect the permeability of the outer mitochondrial membrane [230,324], underlying the role of ROS boosting anti-cancer therapies (Table 1) in mediating apoptosis.

Due to the pro-survival role of autophagy, agents targeting this pathway have been explored for their application as an anti-cancer therapeutic strategy. However, there are only a few clinically available modulators of autophagy (Table 4) [325,326,327,328]. Among these, chloroquine (CQ) and its derivate hydroxychloroquine (HCQ) inhibit lysosomal acidification, preventing autophagosome degradation. HCQ showed better results in the clinical trials, including less toxicity than CQ, and is currently being investigated in combination with other anti-cancer therapeutics [325,327,329]. Similar to apoptosis induction, ROS have been implicated in the autophagy of cancer cells as well [230,324]. ROS act as signalling molecules mediating survival-prone autophagy. However, an excess of ROS influences autophagic cell death [230,324]. This demonstrates another mechanism of pro-oxidant approaches in anti-cancer treatment (Table 1).

### 4.4. Clinical Potential of Targeting the CSC–TME Feedback Loop

Several approaches for targeting CSCs are currently being investigated. Examples include targeting (i) CSC surface markers, such as CD20 and Cd123; (ii) CSC-associated signalling pathways, such as Wnt, Notch and Hedgehog; (iii) the CSC microenvironment, such as the anti-CXCR4 agent plerixafor and (iv) CSC-directed immunotherapy to checkpoint receptors [331,332,333,334,335,336,337,338,339,340,341,342]. However, the robustness of CSCs and their ability to adapt under stress conditions have made it difficult to target these cells. For example, it was suggested that targeting oxidative phosphorylation with mitochondria-targeting drugs, in combination with conventional chemotherapeutics targeting rapidly proliferating cancer cells that utilise glycolysis, might be a practical approach to treatment [205]. This combination therapy has been shown to deplete intracellular ATP in glioma CSCs and prevent breast and lung cancer tumour growth in vivo [343]. Unfortunately, the clinical utility of these agents was limited due to the adaptation of CSCs to ATP depletion and reduced oxidative phosphorylation [344]. One of the main adaptation mechanisms is facilitated by the AMP-mediated activation of AMPK by LKB1 following a decrease in cellular ATP [344,345]. Adding inhibitors of AMPK to prevent CSC resistance mediated by the compensatory activation of mitophagy and mitochondrial fatty acid oxidation may be useful [192,205,346]. Another potential limitation is the ability of cells to undergo metabolic changes following the inhibition of mitochondrial–metabolic pathways [205,347]. This ability to adapt promotes the selection of highly plastic CSCs capable of switching between proliferative and quiescent states. In order to effectively target CSCs, it appears that multiple approaches must be utilised to limit their ability to adapt under stress.

An interesting approach that has reached clinical trials involves loading CSCs isolated from tumours onto dendritic cells, which are then used as a cancer vaccine (NCT02089919, NCT02074046, NCT02063893). However, targeting CSCs alone is unlikely to be highly effective, given their functional intertwinement with the TME. As discussed, the TME surrounding CSCs creates a niche that supports the survival of CSCs and affects drug sensitivity [224]. Thus, it is advisable to investigate combined approaches targeting CSCs with those targeting cells, which support CSC survival, such as CAFs.

## 5. Conclusions and New Directions for Anti-Cancer Strategies

The TME consists not only of a heterogeneous population of cancer cells but also a variety of resident and infiltrating host cells, secreted factors and ECM proteins. Here, we have highlighted how tumour progression is profoundly influenced by the close interaction of cancer cells with their TME and how the microenvironmental stress significantly contributes to tumour development and progression. We have also discussed the vital role the TME has in shaping the therapeutic responses and development of drug resistance. Indeed, targeting TME, as well as its components, offers a promising strategy to overcome drug resistance and treat cancer.

Overall, conventional anti-cancer drugs promote stress in the TME, which enhances and selects for MDR CSCs. In fact, CSCs have been shown to rapidly develop adaptive mechanisms that allow them to not only survive but thrive within a stressful TME. Thus, the TME stress drives and maintains cancer stemness and promotes an MDR phenotype typical for refractory cancers. We propose that strategies reducing microenvironment stress warrant further research as they might diminish the “ready-to-act” state of the cancer cells/CSCs and paradoxically pre-sensitise them to conventional therapy (Figure 3).

## Figures and Tables

**Table 1:** ROS-modulating agents explored in cancer clinical trials.

ROS Modulating Strategies	Compounds Involved in Cancer Clinical Trials
***Antioxidant approach***	
intake of antioxidants	vitamins A [231], C [232] [233] and E [234], selenium [235]
NADPH oxidase inhibition	histamine [238]
GSH induction	sulforaphane [236,237]
nitroxide compound manipulation	tempol [239]
***Pro-oxidant approach***	
ROS generation	arsenic trioxide [249], imexon [248], doxorubicin, daunorubicin [250], cisplatin, oxaliplatin [251], sunitinib [252], gefitinib, erlotinib [253], trastuzumab [254], bevacizumab [255]
GSH depletion	β-phenylethyl isotiocyanate [241], buthionine sulfoximine [242]
thioredoxin inhibition	PX-12 [243], motexafin gadolinium [244]
superoxide dismutase inhibition	2-methoxyestradiol [245], ATN-224 [246], disulfiram [247]

**Table 2:** An example of agents targeting the HIF-1α pathway that have been tested in clinical trials.

Mechanism of Action	Compounds Involved in Cancer Clinical Trials
inhibition of HIF-1α mRNA expression	aminoflavone [257]
inhibition of HIF-1α protein synthesis	topotecan [261], irinotecan [260], EZN-2208 [259], temsirolimus [263], everolimus [262], sirolimus [264], LY294002 [265], digoxin [258], 2-methoxyestradiol [266]
inhibition of HIF-1α stabilisation	geldanamycins [268], SCH66336 [267], apigenin [269], romidepsin [270]
inhibition of HIF-1α dimerisation	acriflavine [271]
inhibition of HIF/DNA binding	doxorubicin, daunorubicin, epirubicin [272]
inhibition of HIF-1 transcriptional activity	bortezomib [273]
inhibition of HIF-1α at multiple levels	PX-478 [274], glycyrrhizin [277,278,279], licochalcone A [275]
HIF-1α degradation	vorinostat [276]

**Table 4:** Compounds targeting apoptosis and autophagy explored in clinical trials.

Apoptosis and Autophagy Targeting Approaches	Compounds Involved in Cancer Clinical Trials
***Stimulating the pro-apoptotic molecules***	
BAX activator	quercetin [309], annonacin [310]
BAX upregulation	thioridazine [205]
DR4 agonist	mapatumumab [312]
DR5 agonist	conatumumab [313], lexatumumab [315], tigatuzumab [314]
DR4/5 agonist	dulanermin [311]
***Inhibiting the anti-apoptotic molecules***	
Bcl-2 antagonist	ABT-737 [319], navitoclax [318], venetoclax [317], AT101 [205,330], curcumin [320], annonacin [310]
Bcl-2 downregulation	thioridazine [205]
IAP antagonist	AT-406, birinapant [316], GDC-0917 [321], LCL161 [322]
XIAP antagonist	curcumin [320]
XIAP antisense oligonucleotide	AEG35156 [323]
***Inhibiting autophagy***	
autophagosome formation inhibition	SF1126 [328], verteporfin [326]
targeting lysosomes	chloroquine [327], hydroxychloroquine [325]

BAX, Bcl-associated X protein; Bcl-2, B-cell lymphoma 2; DR, death receptor; IAP, inhibitor of apoptosis protein; XIAP, X-linked inhibitor of apoptosis protein.
